# Treatment of Catheter-Associated Internal Jugular Vein Thrombosis Using Apixaban for Less Than Three Months in Two Patients With Aggressive B-cell Lymphoma Undergoing Rituximab, Cyclophosphamide, Doxorubicin, Vincristine, and Prednisolone Therapy

**DOI:** 10.7759/cureus.58528

**Published:** 2024-04-18

**Authors:** Takuya Matsunaga, Hiroyuki Kita, Kazuyuki Naito, Masako Morimoto, Katsuya Nakanishi

**Affiliations:** 1 Department of Hematology, Japan Community Health Care Organization (JCHO) Sapporo Hokushin Hospital, Sapporo, JPN; 2 Department of Cardiology, Japan Community Health Care Organization (JCHO) Sapporo Hokushin Hospital, Sapporo, JPN; 3 Department of Cardiology, Sapporo Central Hospital, Sapporo, JPN; 4 Department of Pharmaceuticals, Japan Community Health Care Organization (JCHO) Sapporo Hokushin Hospital, Sapporo, JPN; 5 Department of Pathology, Japan Community Health Care Organization (JCHO) Sapporo Hokushin Hospital, Sapporo, JPN

**Keywords:** malignant lymphoma, apixaban, catheter-associated thrombosis, central venous catheters, internal jugular vein thrombosis

## Abstract

The selection of anticoagulant therapy and appropriate duration of treatment for central venous (CV) catheter-associated internal jugular vein thrombosis in patients with malignant lymphoma remain unclear. Two cases of aggressive B-cell lymphomas treated with R-CHOP (rituximab, cyclophosphamide, doxorubicin, vincristine, and prednisolone), in which apixaban administered for less than three months was effective against CV catheter-associated internal jugular vein thrombosis, are reported. In one case, the right internal jugular vein thrombosis developed after eight courses of R-CHOP; when apixaban was orally administered for 37 days after the CV catheter was removed, the thrombus completely dissolved and did not recur for 27 months. In the other case, right internal jugular vein thrombosis developed after four courses of R-CHOP; two additional courses of the R-CHOP were administered alongside oral apixaban administration without catheter removal. After 66 days of oral apixaban, the thrombus completely dissolved, the CV catheter was removed, and no recurrence was observed for 8.5 months.

## Introduction

Central venous (CV) catheters are commonly used to safely administer chemotherapy to patients with cancer, including those with malignant lymphomas. However, patients with cancer, including those with hematological malignancies, are susceptible to CV catheter-associated thrombosis due to their hypercoagulable state [1-6]. The internal jugular vein is one of the routes used for CV catheter insertion; however, it is also one of the most frequent sites of CV catheter-associated deep venous thrombosis (DVT) [7]. Without prompt treatment, this condition can lead to catheter dysfunction, requiring suspension of chemotherapy; in rare cases, it can also lead to pulmonary embolism.

Low molecular weight heparin (LMWH) and warfarin are the most frequently used treatments for venous thromboembolism (VTE). Factor Xa inhibitors have recently been introduced as an alternative to LMWH and warfarin treatment. Clinical trials have compared the therapeutic effect of factor Xa inhibitors and LMWH for treating cancer-related VTE [8-11]. In these trials, factor Xa inhibitors were continuously administered for over six months; findings revealed that the rates of VTE recurrence and bleeding (as side effects) with factor Xa inhibitors were equivalent to or not higher than those with an LMWH. However, to our knowledge, no comparative trials of factor Xa inhibitors and LMWH have been conducted on the treatment of CV catheter-associated internal jugular vein DVT in patients with lymphomas.

Furthermore, few comparative trials have been conducted on the treatment of CV catheter-associated upper extremity DVT (UEDVT) in patients with cancer. Guidelines on the treatment of VTE recommend that LMWH be administered for at least three months as the first-line treatment for CV catheter-associated UEDVT in patients with cancer [12-14]. However, this recommendation is based on studies of UEDVT and DVT of the lower extremities conducted in patients without cancer [15].

Currently, no guidelines are available on the treatment of CV catheter-associated internal jugular vein DVT in patients with hematological malignancies. Therefore, the most effective treatment option and appropriate duration of treatment for anticoagulation of CV catheter-associated internal jugular vein DVT in patients with malignant lymphoma remain unclear. Therefore, to contribute to the accumulation of evidence, we present two cases of patients with aggressive B-cell lymphoma treated with R-CHOP (rituximab, cyclophosphamide, doxorubicin, vincristine, and prednisolone), in which apixaban administered for less than three months was effective against CV catheter-associated internal jugular DVT.

## Case presentation

Case 1

In January 2021, a 72-year-old man with a fever was referred to our Department of General and Emergency Medicine from another hospital. At presentation, the patient was febrile, with no abnormal findings on physical examination. Computed tomography (CT) revealed mediastinal lymphadenopathy (tracheal bifurcation, anterior mediastinal, and paratracheal lymph nodes) and mild enlargement of the abdominal periaortic lymph nodes. A diagnosis of follicular lymphoma (grade 3B) was established based on cells collected from the enlarged tracheal bifurcation lymph nodes via endobronchial ultrasound-guided transbronchial needle aspiration. The patient was then referred to the Department of Hematology. Endoscopy of the upper and lower gastrointestinal tract revealed no lymphoma lesions. However, bone marrow biopsy from the posterior superior iliac crest showed lymphoma cell infiltration of the bone marrow. The patient was determined to have stage IV disease, per the Lugano classification system. The serum levels of C-reactive protein (CRP), lactate dehydrogenase (LDH), and soluble interleukin-2 receptor (sIL-2R) and D-dimer were increased (Table 1). However, the serum albumin level (Alb) and the estimated glomerular filtration rate (eGFR) were decreased. The serum creatinine (Cr) was within the normal range.

**Table 1:** Initial laboratory results in Case 1 Relevant laboratory tests include complete blood count, blood chemistry test, and sIL-2R. BUN: blood urea nitrogen; CRP: C-reactive protein; LDH: lactate dehydrogenase; sIL-2R: soluble interleukin-2 receptor; eGFR: estimated glomerular filtration rate

Parameter	Value	Reference values
Leukocyte count	5.99x10^3^/uL	3.5-9.7x10^3^/uL
Hemoglobin	12.9 g/dL	12-18 g/dL
Hematocrit	41.7%	40-52%
Platelets	360.8x10^3^/uL	140-380x10^3^/uL
CRP	15.66 mg/dL	0.00-0.30 mg/dL
LDH	428 U/L	120-245 U/L
sIL-2R	1,293 U/ML	122-496 U/ML
Serum albumin	3.1 g/dL	3.8-5.2 g/dL
BUN	16.8 mg/dL	8-20 mg/dL
Serum creatinine	0.79 mg/dL	0.46-0.82 mg/dL
eGFR	73.9 mL/min/L	≥89.0 mL/min/L
D-dimer	2.18 ug/mL	0.00-1.00 ug/mL

**Figure 1:**
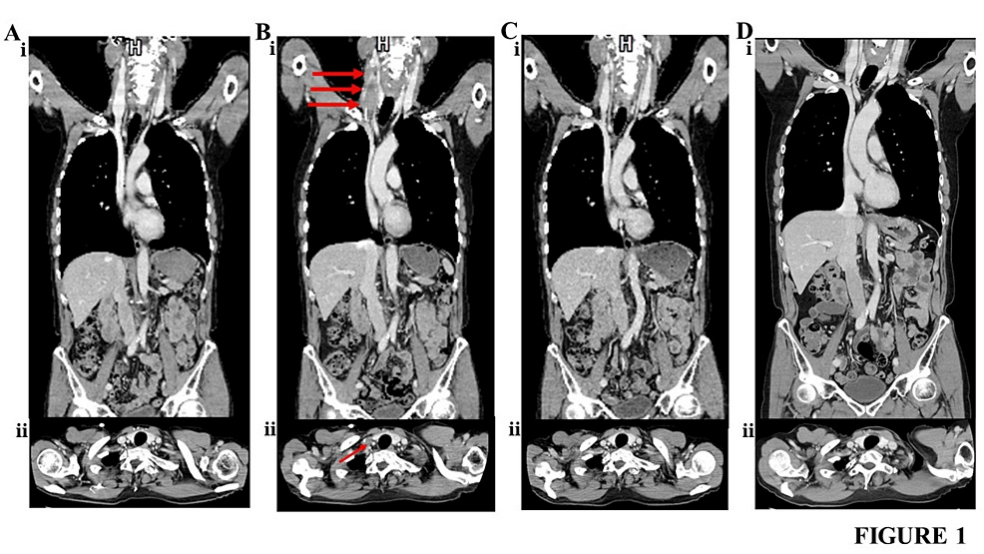
Contrast-enhanced CT findings in Case 1 (A) CT findings obtained on July 29, 2021. (i) Systemic findings and (ii) sixth cervical vertebrae findings. (B) CT findings obtained on August 20, 2021. (i) Systemic findings and (ii) sixth cervical vertebrae findings. (C) CT findings obtained on September 25, 2021. (i) Systemic findings and (ii) sixth cervical vertebrae findings. (D) CT findings obtained on January 4, 2024. (i) Systemic findings and (ii) sixth cervical vertebrae findings. Arrows indicate the thrombus in the right internal jugular vein at the CV catheter insertion site in the CV port. CT: computed tomography; CV: central venous

The patient underwent CV port placement for chemotherapy. The first course of R-CHOP was initiated on February 12, 2021, followed by seven more courses. Contrast-enhanced CT, performed on July 29, 2021 (i.e., after the seventh course of the R-CHOP), showed no thrombi in the internal jugular vein (Figure 1A). The eighth course of R-CHOP was initiated on August 4, 2021. On August 20, 2021, an elevated CRP level (2.62 mg/dL) and slight swelling on the right side of the neck were observed. Contrast-enhanced CT revealed a thrombus around the CV catheter, almost occluding the internal jugular vein (Figure 1B).

A complete response was observed after the originally planned eight courses of the R-CHOP; therefore, we decided to terminate chemotherapy and immediately removed the CV port and CV catheter. Placement of the superior vena cava filter before the removal of the CV catheter may be considered an option. Apixaban was initiated thereafter. During the first week, 10 mg was administered twice daily; thereafter, 5 mg was administered twice daily. We determined the dosage of apixaban according to the package insert of apixaban in Japan. Contrast-enhanced CT, performed on September 25, 2021 (i.e., 37 days after the start of the apixaban treatment), revealed complete disappearance of the right internal jugular vein thrombus; thus, apixaban was discontinued (Figure 1C). D-dimer was reduced to within the reference value (0.45 ug/mL). Incidentally, no adverse events involving bleeding, such as gastrointestinal bleeding, were observed during apixaban administration. Contrast-enhanced CT, performed on January 4, 2024, revealed no recurrence of the right internal jugular vein thrombus (Figure 1D); this recurrence-free state had been maintained for 27 months until then.

Case 2

In October 2022, a 68-year-old woman with a diffuse large B-cell lymphoma (DLBCL) of the left nasal cavity was referred to our Department of Hematology from the Department of Otolaryngology at another hospital. At presentation, she was afebrile, and no abnormal findings were found on physical examination. CT revealed a soft tissue concentrated mass measuring >4 cm in the left lower nasal passage. Endoscopy of the upper and lower gastrointestinal tract showed no lymphoma lesions. Bone marrow biopsy from the posterior superior iliac crest showed no bone marrow infiltration of lymphoma cells. Positron emission tomography-CT (PET-CT) revealed fluorodeoxyglucose accumulation in the left nasal cavity, consistent with the DLBCL in this region. Accordingly, a diagnosis of stage I disease was established based on the Lugano classification system. The eGFR and the serum levels of CRP, LDH, sIL-2R, Alb, Cr, and D-dimer were within their normal ranges (Table 2).

**Table 2:** Initial laboratory results in Case 2 Relevant laboratory tests include complete blood count, blood chemistry test, and sIL-2R. BUN: blood urea nitrogen; CRP: C-reactive protein; LDH: lactate dehydrogenase; sIL-2R: soluble interleukin-2 receptor; eGFR: estimated glomerular filtration rate

Parameter	Value	Reference values
Leukocyte count	5.74x10^3^/uL	3.5-9.7x10^3^/uL
Hemoglobin	13.9 g/dL	12-18 g/dL
Hematocrit	41.5%	40-52%
Platelets	370.2x10^3^/uL	140-380x10^3^/uL
CRP	0.13 mg/dL	0.00-0.30 mg/dL
LDH	200 U/L	120-245 U/L
sIL-2R	275 U/ML	122-496 U/ML
Serum albumin	4.4 g/dL	3.8-5.2 g/dL
BUN	15.3 mg/dL	8-20 mg/dL
Serum creatinine	0.39 mg/dL	0.46-0.82 mg/dL
eGFR	120.3 mL/min/L	≥89.0 mL/min/L
D-dimer	0.71 ug/mL	0.00-1.00 ug/mL

**Figure 2:**
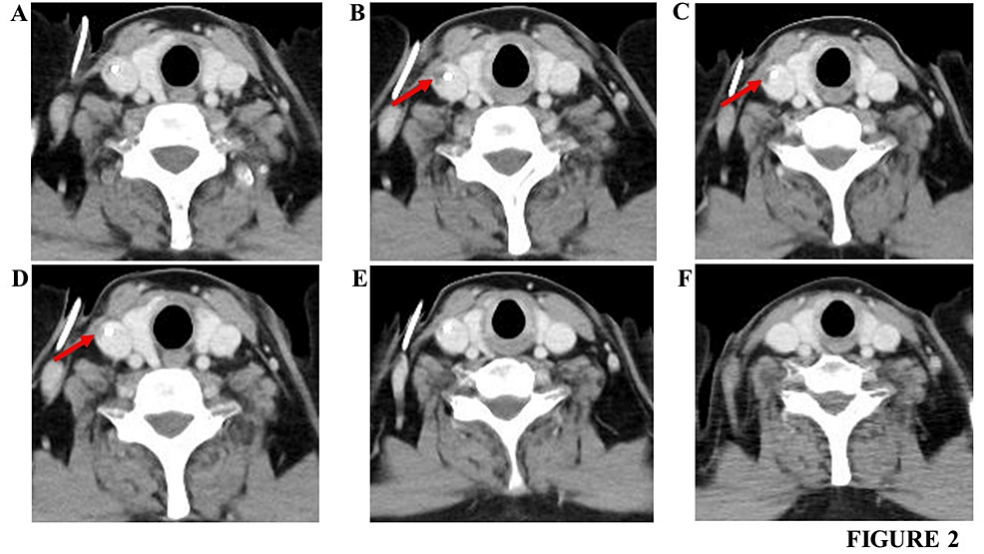
Contrast-enhanced CT findings of the sixth cervical vertebrae in Case 2 (A) CT findings obtained on December 7, 2022. (B) CT findings obtained on January 24, 2023. (C) CT findings obtained on February 2, 2023. (D) CT findings obtained on February 20, 2023. (E) CT findings obtained on March 13, 2023. (F) CT findings obtained on December 4, 2023. Arrows indicate the thrombus in the right internal jugular vein at the CV catheter insertion site in the CV port. CT: computed tomography; CV: central venous

The patient underwent CV port placement for chemotherapy. The first course of the R-CHOP was initiated on November 1, 2022. This regimen was continued for the next three courses. Contrast-enhanced CT, performed on December 7, 2021 (i.e., after the second course of the R-CHOP), showed no thrombi in the internal jugular vein (Figure 2A). On January 24, 2023, contrast-enhanced CT was performed to evaluate the effect of the fourth course of R-CHOP. Although a complete response was observed, a 7-mm contrast defect was noted in the right internal jugular vein at the CV catheter insertion site; thus, the patient was diagnosed with a thrombus (Figure 2B). D-dimer was within the reference value (0.88 ug/mL).

The original treatment plan involved a minimum of six courses of R-CHOP. Thus, apixaban was immediately initiated without removing the CV port (with the CV catheter) since the CV catheter was functioning and continuous catheter use was required. During the first week, 10 mg was administered twice daily, and thereafter, 5 mg was administered twice daily. We determined the dosage of apixaban according to the package insert of apixaban in Japan. Thus, two more courses of R-CHOP were administered with oral apixaban without catheter removal. On February 2, 2023, contrast-enhanced CT was performed to evaluate the effect of apixaban. A 5-mm contrast defect was observed in the right internal jugular vein at the CV catheter insertion site, and the thrombus appeared to have shrunk slightly (Figure 2C). On February 20, 2023 (i.e., after the fifth course of the R-CHOP), contrast-enhanced CT revealed a 2-mm contrast defect in the right internal jugular vein at the CV catheter insertion site; the thrombus had shrunk further (Figure 2D). On March 13, 2023 (i.e., after the sixth course of the R-CHOP), contrast-enhanced CT revealed no contrast defect in the right internal jugular vein at the CV catheter insertion site; the thrombus had dissolved completely (Figure 2E). On March 30, 2023, PET-CT (performed to evaluate the effect of the six R-CHOP courses) revealed a complete response. Therefore, the CV catheter was removed 66 days after oral apixaban initiation, and apixaban was discontinued. Incidentally, no adverse events involving bleeding, such as gastrointestinal bleeding, were observed during apixaban administration. Contrast-enhanced CT, performed on December 4, 2023, revealed no recurrence of the right internal jugular vein thrombus (Figure 2F); this recurrence-free state had been maintained for 8.5 months until then.

## Discussion

Patients with malignancies are at an increased risk of thrombosis, especially after CV catheter placement [1-6]. In patients with malignant lymphoma, anthracycline-containing chemotherapy regimens further increase the risk of CV catheter-associated DVT [5]. This is supported by the fact that both Cases 1 and 2 presented herein were treated with the R-CHOP, which includes the anthracycline anticancer drug doxorubicin.

We administered oral apixaban in an attempt to dissolve the CV catheter-related right internal jugular vein thrombus in both cases. No treatment has been established for CV catheter-associated DVT in hematological malignancies. Furthermore, no reports are available on the selection of drugs for anticoagulation therapy of catheter-associated internal jugular vein DVT in patients with malignant lymphomas. VTE treatment guidelines recommend LMWH as the first-line anticoagulant for treating VTE in patients with cancer [12-14]. The results of trials comparing factor Xa inhibitors with LMWH for treating VTE in patients with cancer have shown that factor Xa inhibitors are acceptable as an alternative therapeutic option [12,13,16]. Then again, in these trials, patients with malignant lymphomas accounted for only 5-10% of the sample population [8-11]. Therefore, insufficient data are available on the relative safety and effectiveness of factor Xa inhibitors and LMWH for treating VTE in patients with malignant lymphomas. Furthermore, randomized controlled trials have not been conducted on different factor Xa inhibitors for treating cancer-related VTE. We decided to use apixaban, which is easier to administer than LMWH; furthermore, we inferred that apixaban had a lower bleeding risk compared to rivaroxaban and edoxaban.

Various guidelines recommend the administration of anticoagulation therapy for at least three months to treat cancer-related VTE [12-14,16]. However, insufficient research has been conducted on the appropriate duration of anticoagulation therapy. The 2012 guidelines for UEDVT by the American College of Chest Physicians have provided the following recommendations. First, in cancer patients with CV catheter-associated UEDVT, the catheter should not be removed if it is functioning and if continuous catheter use is required. Second, in cancer patients with UEDVT who had their CV catheter removed, anticoagulant administration should be continued for at least three months. Finally, in cancer patients with UEDVT who had not had their CV catheter removed, anticoagulation therapy should be administered for a minimum of three months and should be continued for as long as the CV catheter remains in place [17]. In Case 1, the patient had an internal jugular vein thrombus, and the CV catheter was removed. Apixaban was discontinued after 37 days because the thrombus had completely dissolved. In Case 2, the patient had an internal jugular vein thrombus, and the CV catheter remained in situ. Apixaban was discontinued after 66 days because the thrombus had completely dissolved. In both cases, long-term follow-up confirmed a lack of recurrence of internal jugular vein thrombosis. To our knowledge, no reports are available on the appropriate duration of apixaban therapy for treating catheter-associated internal jugular vein DVT in patients with malignant lymphomas. In both our cases of aggressive B-cell lymphomas treated with R-CHOP, apixaban was administered for less than three months and was effective against CV catheter-associated internal jugular vein DVT. The D-dimer in Case 1 was elevated before the start of R-CHOP and decreased to within the reference value after thrombolysis. On the other hand, the D-dimer in Case 2 was within the reference values both before the start of R-CHOP and at the time of detection of the thrombus. These data do not provide evidence that D-dimer is useful in predicting the occurrence of catheter-associated internal jugular vein thrombosis or in determining the effectiveness of treatment. In patients with malignant lymphomas, the remission status may be an important determinant of CV catheter-associated DVT recurrence. In cases wherein patients exhibit a complete response to treatment (as in our two cases), CV catheter-associated DVT may have developed independent of the malignant lymphoma, and in such cases, the VTE recurrence rate is likely to be low.

There are two limitations in our case series. First, we provide empirical evidence only on the two cases presented. Second, retrospective analyses were performed in this case series. In the future, we plan to conduct prospective research to address these limitations.

## Conclusions

This is the first report to suggest that less than three months of apixaban administration is effective against CV catheter-associated internal jugular vein thrombosis in aggressive B-cell lymphoma patients treated with R-CHOP. The thrombi in Cases 1 and 2 completely dissolved and did not recur for 27 and 8.5 months after treatment, respectively. However, future prospective clinical trials are required to validate our observations.
